# The Relationship between the Length/Width of the Face and the Length/Width of the Crown of the Permanent Upper Central Incisors

**DOI:** 10.3390/jcm13164698

**Published:** 2024-08-10

**Authors:** Tilen Dervarič, Anita Fekonja

**Affiliations:** 1 Faculty of Medicine, University of Maribor, Taborska Ulica 8, 2000 Maribor, Slovenia; tilen.dervaric@student.um.si; 2 Community Health Centre Maribor, Department of Orthodontic, Ulica Talcev 9, 2000 Maribor, Slovenia

**Keywords:** face dimensions, upper central incisors, facial index, tooth index

## Abstract

**Background:** The face, with its attractiveness, is positively connected with self-esteem and interpersonal relationships, and the maxillary central incisors are the most noticeable teeth and crucial for smile aesthetics. The aim of this study is to examine facial and permanent upper central incisors’ clinical crown dimensions and their correlations to establish whether there is a relationship between the length/width of the face and the length/width of the clinical crown of the permanent upper central incisors. **Methods:** This study included 100 subjects (43 males and 57 females) with a mean age of 17.5 ± 3.4 years before orthodontic treatment. Facial length and width were measured using a cephalometer by the same orthodontist and the upper central incisors’ clinical crown lengths and widths were measured using a sliding calliper by the same dental student. Data were analysed using SPSS version 29.0, presenting descriptive statistics for age, facial and upper central incisor crown dimensions, and indices. Pearson’s correlation coefficient assessed the relationship between facial features and the upper central incisors’ crown. The significance level was *p* ≤ 0.05. **Results:** Males exhibit a slightly higher mean value of the face length (11.6 ± 0.8 cm) compared to females’ face length (11.2 ± 1.1 cm) and a statistically significant (*p* < 0.05) higher mean value of the face width (11.7 ± 0.8 cm) compared to females (11.1 ± 0.6 cm). The facial index shows no statistically significant gender difference (males: 99.1 ± 8.4; females: 101.2 ± 11.9, *p* > 0.05). The upper central incisor crown dimensions are without statistically significant differences between gender and tooth side: males have mean value clinical crown lengths of 10.2 ± 1.1 mm (left) and 10.1 mm ± 1.2 (right) while females have 9.9 ± 1.0 mm (left) and 9.8 ± 0.7 mm (right). Clinical crown widths are also comparable (males: left 9.0 ± 0.7, right 8.9 ± 0.6 and females: left 8.8 ± 0.7, right 8.8 ± 0.7), with no statistically significant differences observed. The tooth index shows no statistically significant difference but there is a statistically significant (*p* < 0.05) correlation between the facial index and tooth index. **Conclusions:** Knowledge of the presented data is important for diagnosis and treatment planning and may be useful for restorative dentists, orthodontists, prosthodontists, and maxillofacial surgeons to achieve an aesthetic result. Clinically, our study supports the development of more personalized treatment plans.

## 1. Introduction

The upper central incisors are the most noticeable teeth in the mouth, and when smiling, the upper central incisors become the key point for creating the illusion that they are the brightest and largest teeth in the mouth [1,2]. Understanding their aesthetic perception is crucial for treatment planning, particularly in determining the ideal width-to-height ratio to achieve a satisfactory aesthetic result. For example, in cases of missing or worn central incisors, accurate measurements can aid in orthodontic or restorative treatment. Various proportions, such as the Golden Proportion and the width-to-height ratio of the maxillary anterior teeth, are often used in aesthetic rehabilitation [3].

The upper central incisors have specific dimensions and shapes. Typically, they are 8–9 mm wide and 10–11 mm long. Their labial surface is less convex than other teeth in the intercanine area, giving them a rectangular appearance. The incisal edge is straight, the mesial incisal angle is sharp, and the distal incisal angle is rounded [3]. Research has shown differences in the size and shape of anterior teeth among different races and genders. These findings are important for restorative treatment plans to achieve optimal aesthetic and functional results [4,5,6,7].

Studies of facial proportions have revealed various factors that determine attractiveness, including facial dimensions, symmetry, and sexual dimorphism. Patient’s appearance preferences and muscle movements that express emotions are also considered. However, despite studies, it remains unclear how different factors of orofacial harmony affect the perception of smile attractiveness [8,9].

Several researchers observed the harmony of the shape of the upper central incisors and the outline of the face. For an aesthetically pleasing appearance, it is important that the teeth are in proportion to the outline of the face. The optimal width-to-length ratio of the upper central incisors’ crown ranges from 66% to 85%, with the ideal being approximately 80% [10,11,12,13,14].

Understanding facial measurements is crucial for aesthetic procedures and forensic medicine as it allows for the adaptation of reconstructive surgeries to achieve symmetrical and aesthetically pleasing results [15,16,17,18].

We conducted this study to gain a deeper understanding of the aesthetic characteristics of the upper central incisors in the local population of the Maribor area. This study had two main goals: first, to establish baseline measurements for face length and width as well as the clinical crown length and width of the upper central incisors in this demographic; second, to analyse the relationship between the facial index and the tooth index. By identifying patterns and correlations, we aim to inform more personalized and effective restorative dental, orthodontic, and prosthodontic treatments.

## 2. Materials and Methods

Ethical approval for this study was obtained from the Institutional Review Board at the Community Healthcare Centre Maribor, Slovenia (02/010/03-10/01/24). This study was conducted in accordance with the Declaration of Helsinki at the Orthodontic Department of Community Healthcare Centre Maribor. Informed consent was obtained from each subject.

This study used a randomly selected sample of 100 Caucasian subjects before orthodontic treatment with permanent dentition (excluding third molars) aged up to 16 years who had consistent facial proportions without noticeable asymmetry and no dental deformities, mesio-distal and cervico-incisal crown abrasion, restorations extending to the mesial or distal surfaces, enamel stripping of the upper central incisors, gingival recession, or periodontitis. Subjects with syndromes, orofacial developmental anomalies, facial bone injuries, or deformities were excluded to avoid severe craniofacial irregularities that could affect this study’s measurements and outcome.

### 2.1. Facial Measurement

**Figure 1:**
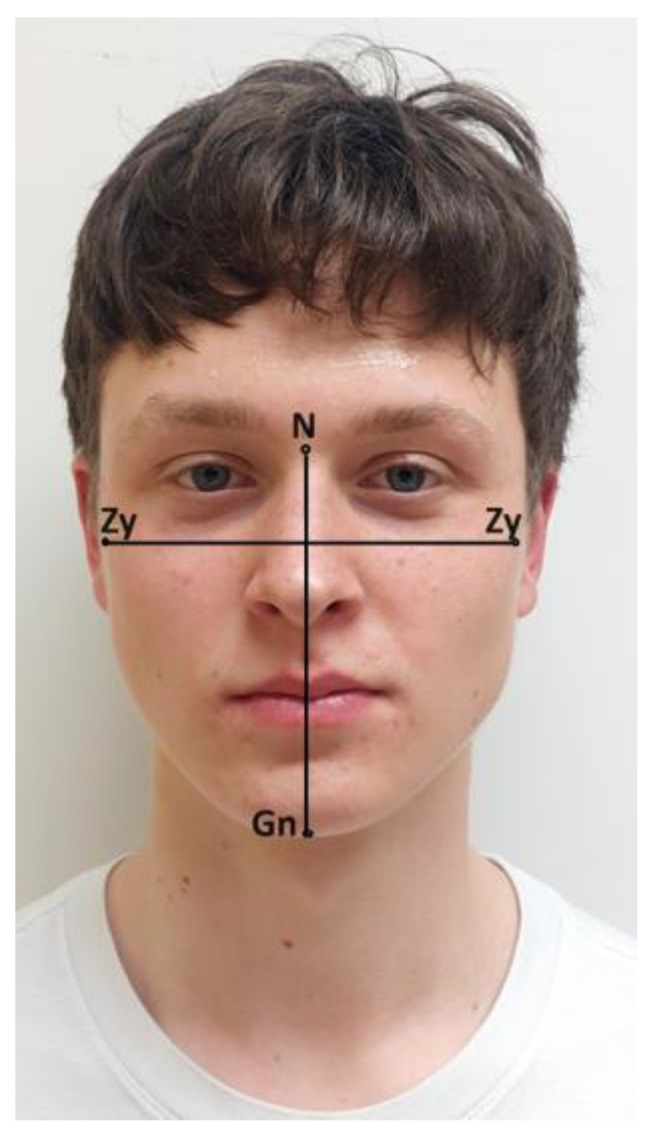
Anthropometric points and linear measured values. Legend: N (nasion): the deepest point of the concavity between the forehead and the soft tissue contour of the nose in the midsagittal plane, Gn (gnathion): the most inferior point of the soft tissue contour of the chin, and Zy (zygoma): the most prominent lateral point of the soft tissue on the zygomatic arch.

Facial length and width were measured by the same orthodontist (A.F.) using a cephalometer (Zanatoprema—Rijeka, Croatia), with an accuracy of 1 mm. The measurements were uniform for all subjects. It is a safe, non-invasive, and repeatable method. The subjects were standing, with their lips joined together and their teeth in the maximal intercuspation (centric occlusion). The landmarks were first identified and marked lightly with a pen before taking the measurements. The definition of the anthropological landmarks of the face and linear measured values used are described and illustrated in Figure 1.

**Figure 2:**
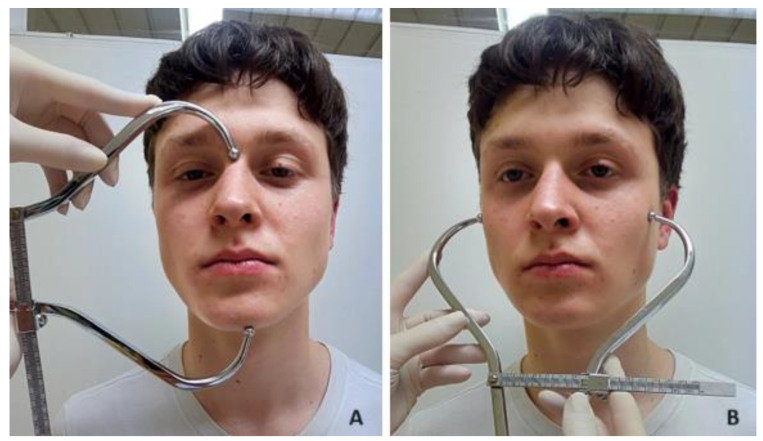
Linear measured values of the face (facial length (**A**) and facial width (**B**)) measured with a cephalometer.

The measured values of the facial dimensions were as follows:

- Facial length was measured as the vertical distance from the nasion to the gnathion (Figure 2A).
- Facial width was measured as the maximum horizontal distance between the lateral borders of the right and left zygoma (Figure 2B).

### 2.2. Dental Measurements

The clinical crown length (cervico-incisal diameter) and clinical crown width (mesio-distal diameter) of the upper central incisors were measured and collected by the same dental student (T.D.) using a sliding calliper (Münchner Modell REF 042-751-00, Dentaurum Germany) with an accuracy of 0.1 mm. The clinical crown length and width were measured from the facial aspect using the outer edges of the sliding calliper. The calliper was held parallel to the occlusal and buccal surfaces.

**Figure 3:**
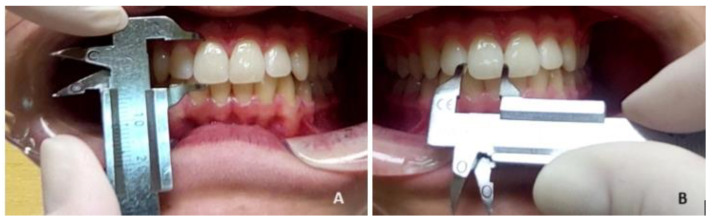
Linear measured values of the maxillary central incisors (length (**A**) and width (**B**)) measured with a vernier calliper.

The measured values of the clinical crown dimensions were as follows:

- The clinical crown length was measured as the greatest vertical distance between the most apical point of the cervical line of the marginal gingiva and the incisal edge (Figure 3A).
- The clinical crown width was measured as the widest distance between the mesial and distal contact points on the interproximal surfaces of the tooth crown (Figure 3B).

**Figure 4:**
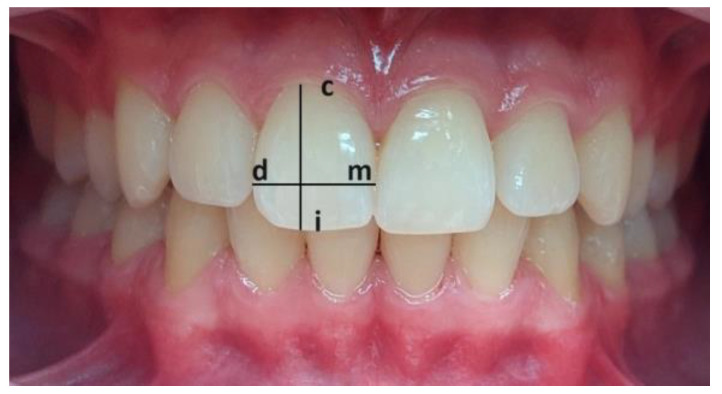
Points and linear measurements. Legend: c: the most apical point of the cervical line of the marginal gingiva, d: distal contact point on the interproximal surface of the clinical crown, m: mesial contact point on the interproximal surface of the clinical crown, and i: the incisal edge.

The definition of the landmarks and the linear measured values used are described and illustrated in Figure 4.

### 2.3. Facial Index

The facial index was calculated as a ratio between facial length and facial width using the facial index formula (Equation (1)). Faces can be classified into three types using the facial index (Table 1) [19].

(1) $$Facial\;index=\frac{Facial\;length}{Facial\;width}\times 100$$

Equation (1). Facial index formula.

### 2.4. Tooth Index

The tooth index was calculated as a ratio between tooth width and tooth length using the tooth index formula (Equation (2)) [14].

(2) $$Tooth\;index=\frac{Tooth\;width}{Tooth\;lenght}\times 100$$

Equation (2). Tooth index formula.

#### Statistical Analysis

The collected data were entered into the Statistical Package for Social Science version 29.0 (SPSS Inc. Chicago, IL, USA). Randomly selected subjects were re-measured one month after the initial measurement to evaluate intra-operator errors. A paired *t*-test was used to perform error analysis. The difference was not statistically significant.

The obtained data were statistically analysed using descriptive statistical methods. A descriptive analysis of categorical (gender) and continuous (age, clinical crown width and length, and facial width and length) variables was performed to calculate the frequency, percentage, mean value, and standard deviation. Moreover, regression analysis, independent *t*-test, and paired *t*-test were used to compare the mean values of dependent (face width and length and tooth clinical crown width and length) and independent (age and gender) variables. A *p* value of ≤0.05 was considered statistically significant. We also used the correlation coefficient, which ranges from −1 (indicating a perfect negative correlation) to 1 (indicating a perfect positive correlation), with 0 representing no linear correlation.

## 3. Results

A total of 100 subjects (43 males and 57 females) with a mean age of 17.5 years (standard deviation of 3.4 years) were included in this study. There was no statistically significant difference (*p* > 0.05) between gender and age. Descriptive statistics are presented in Table 2.

The anthropometric measured values of the face according to gender are presented in Table 3. This intergender comparison shows that male and female faces differ regarding measurements. Males tend to have a higher mean value of the face length than females, with this being close to statistical significance (*p* = 0.071). The face width measured values reveal a statistically significant difference (*p* < 0.05), with males having a statistically significant higher mean value of the face width compared to females. The facial index, which assesses the ratio between facial length and width, showed no statistically significant difference between genders (*p* > 0.05).

The results of the face type distribution between genders are presented in Table 4. The most common face type is the leptoprosopic face in both genders. Mesoprosopic and euryprosopic faces are presented to a smaller extent. No statistically significant difference (*p* > 0.05) was observed between face type and gender (Table 5).

The results of the clinical crown dimension measurements (length and width) are presented in Table 6 and show the differences between the mean value of the clinical crown length and width on the left and right sides according to gender, but there were no statistically significant differences (*p* > 0.05).

The results of a statistically significant (*p* < 0.05) correlation between the tooth index and the facial index according to gender are presented in Table 7.

**Figure 5:**
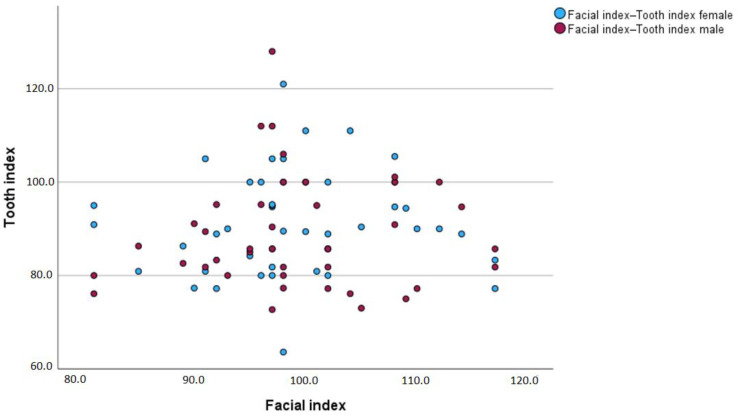
Correlation between tooth index and facial index according to gender.

Figure 5 illustrates the relationship between the facial index and the tooth index according to gender. The graph shows a wide spread of data points without a clear linear trend, suggesting that there is no strong correlation between the facial index and the tooth index for either gender. The clustering of data points around similar facial index values indicates that the tooth index varies independently of the facial index in males and females.

Additionally, the overlapping of red and blue dots implies that both genders exhibit a similar range of tooth index values across the spectrum of facial index values. This visual representation aligns with the statistical results from Table 7, which shows significant but small differences in the mean tooth index relative to the facial index between males and females.

## 4. Discussion

The knowledge of the anthropometric characteristics of the face and teeth is important for several dental and medical procedures. The face is one of the most diverse parts of the human body. Several studies in the literature have reported that measured face values vary with geographical location, age, and gender. There are heritable differences in the anthropometric characteristics of different populations [20,21,22,23,24,25,26,27,28]. Therefore, it is important to have knowledge of the anthropometric characteristics of a given population.

Although the anthropometric characteristics of the face and upper central incisors have been reported in many countries, there has been no published study of the correlation between the facial index and tooth index in the Slovenian population, part of Central Europe. In the literature, there are studies of head width, length, and height [29,30] and the influence of edentulism on the facial characteristics of older adults [31].

The present study included 100 subjects of both genders with permanent dentition (excluding third molars) aged up to 16 years. Subjects with dental deformities, upper central incisor shape change, noticeable facial asymmetry, syndromes, orofacial developmental anomalies, facial bone injuries, or deformities were excluded from this study to avoid irregularities that could affect this study’s measurements and outcome. This homogeneous study group ensured the relevance of the data.

In the present study, the mean values of facial length in males and females were 11.6 ± 0.8 cm and 11.2 ± 1.1 cm, respectively, and the mean values of facial width in males and females were 11.7 ± 0.8 cm and 11.1 ± 0.6 cm, respectively. By comparing the results of the present study with other studies [20,21,22,23,24], differences are evident. Arslan et al. [20], Omotoso et al. [21], and Al-Isheakli [22] reported higher mean values of facial length and width in both genders compared to the results of the present study but without statistically significant differences. Wadud et al. [23] found the highest mean values of facial width in both genders (13.38 cm), which is statistically significantly higher than in the present study. Additionally, Kassab [24] reported lower mean values of facial length and width in females and higher mean values in males compared to our results. In agreement with the results of other authors [20,21,22,23,24], in the present study, males were found to have a higher mean value of facial length and width than their female counterparts. We noted a statistically significant difference (*p* < 0.05) only in facial width, indicating a higher facial width in males compared to females.

The facial index, which measures the ratio of facial length to width, was reported by many researchers [20,21,22,23,24,25,26]. In the present study, the most common facial type identified across both genders was a leptoprosopic face in 86% of subjects, followed by a mesoprosopic face in 9% of subjects and a euryprosopic face in 5% of subjects. Arslan et al. [20] found that in their (Turkish) population, 37.6% of individuals had a leptoprosopic face, 34.1% of individuals had a euryprosopic face, and 28.3% of individuals had a mesoprosopic face. This shows similarities to our study in the presence of different facial types but with a different distribution. Kassab [24] studied an Iranian population, Rokaya et al. [25] studied a Nepali population, and Wadud et al. [23] studied a Thai population. They also reported that the leptoprosopic face was the most common, present in 80%, 69%, and 82.5% of the populations while the euryprosopic face was present in 16%, 23,3%, and 13.5% of the populations, and the mesoprosopic face in 4%, 7.3%, and 4% of the populations, respectively. Again, we observe similarities in the predominant facial type but with different percentages compared to our sampled population. Al-Isheakli [22] also found that the most common was the leptoprosopic face in both genders (78%), but that was followed by the mesoprosopic face (20%) and then the euryprosopic face (2%). Omotoso et al. [21] studied a Nigerian population and reported that the most common was the mesoprosopic face, present in about 80% of their population. Kanan et al. [26] studied an Indian population and found that the euryprosopic face was predominant, present in 78.1% of individuals, while the mesoprosopic face was present in 18.2% and the leptoprosopic face in 3.7%. This significantly differs from our results, where the euryprosopic face is less represented, and the leptoprosopic face dominates.

Brodbelt et al. [32] reported that the upper central incisors are the most important teeth in the selection of anterior teeth since they are most visible to the casual observer in unstrained facial activity. Regarding teeth proportion, gender variations in the dimension of the anterior teeth have been noted in most racial groups with males and females.

In the present study, the comparison of the clinical crown lengths and widths of the left and right sides showed some minor variations. Sah et al. [13] and Alqahtani et al. [27] reported slight differences in clinical crown lengths and crown widths of the left and right sides but without statistically significant differences compared to the present study. Some researchers [11,13,27,33,34] reported that males exhibit longer and wider upper central incisor crowns than females, which is in agreement with the present study. Tsukiyama et al. [35] reported the mean values of crown length in Asian and Caucasian populations were 11.93 ± 0.8 mm and 11.96 ± 0.7 mm, respectively, while the mean values of width were 8.63 ± 0.6 mm and 9.10 ± 0.6 mm, respectively. The Slovenian population is also Caucasian, but the results of the present study vary from the results reported by Tsukiyama et al. [35]. The noticeable difference was in the mean values of crown lengths due to different measurements. Tsukiyama et al. [35] measured extracted teeth from the incisal edge to the cemento-enamel junction. That is contrary to the present study that measured from the incisal edge to the gingival margin, which led to greater length values.

Various studies were conducted to determine the tooth index and the crown width-to-crown length ratio. In the present study, no statistically significant differences in the tooth index between the left and right upper central incisors and between genders were found. Similar findings were published by Hasanreisoglu et al. [11], Sah et al. [13], Alqahtani et al. [27], and Orozco-Varo et al. [34]. However, slight differences in the tooth index between populations are evident, suggesting the importance of considering ethnic and geographical variations when interpreting these findings. The study by Tsukiyama et al. [35] found that the tooth index of the upper central incisors in Caucasian populations was 78 ± 3.0, which is in contrast to the present study of the Slovenian population, also Caucasians, due to the previously mentioned difference in the measurement method.

Cinelli et al. [36], in their literature review, found that there are no standard tooth sizes and that they differ by gender with greater width and length in males, which is in agreement with the present study. They also highlight ethnic differences in dental dimensions, with Caucasian populations generally having larger anterior teeth compared to Asian populations, and discuss the variability in facial–tooth ratios and the implications for aesthetic treatments.

Sellen et al. [37] and Kostić et al. [38] studied the correlation between the shape of the face, upper central incisors, and upper dental arch using photographs and plaster models. Sellen et al. [37] reported that the shape of the face and the upper central incisors matched in 22% of the subjects, the shape of the upper central incisors and the upper dental arch matched in 24% of the subjects, and the shape of the face and the upper dental arch matched in 28% of the subjects. Kostić et al. [38] found no statistically significant gender-related differences in the shape of the upper central incisors that matched the shape of the upper dental arch or in the shape of the upper dental arch that matched the shape of the face. Our study is not fully comparable as we did not investigate the relationship of the shape of the upper dental arch. However, Kostić et al. [38] reported a statistically significant difference in the shape of the upper central incisors that matched the shape of the face, which is in agreement with our results.

Data from the present study of upper central incisors and face dimensions confirm the importance of incorporating the relationship between the tooth and facial indices into restorative, orthodontic, and prosthodontic treatment planning to achieve an improved aesthetic outcome. This is especially important when the upper central incisors are missing or edentulous to create prosthetics that more naturally match individual facial features.

However, the present study had some limitations, too. One notable limitation is the relatively small sample size of 100 patients, which was determined by the limited timeframe and the number of patients meeting the inclusion criteria. This study was also restricted to a Caucasian sample prior to orthodontic treatment. Additionally, this study was conducted in a single orthodontic clinic, limiting the number of available participants. Therefore, future studies should consider expanding the research to include multiple clinics in Maribor and other regions across Slovenia to gain a more comprehensive understanding and validate these findings on a broader scale. Although this study may not represent the entire Slovenian population, these findings provide valuable insights into the relationship between facial dimensions and the upper central incisor dimensions in the selected population and are beneficial for restorative dentists, orthodontists, prosthodontists, and maxillofacial surgeons in treatment planning. Another limitation is the manual measurements of linear variables. In our clinical practice, we have started using scanners since May 2024. Future research should utilize 3D scans.

## 5. Conclusions

In conclusion, the results of the present study are important for restorative dentists, orthodontists, prosthodontists, and maxillofacial surgeons in providing optimal treatment planning. They show statistically significant higher mean values of face width in males compared to females and a statistically significant correlation between the tooth index and facial index in both genders. Clinically, our study supports the development of more personalized treatment plans. Future research could expand the understanding of how tooth dimensions and facial proportions affect long-term treatment outcomes.

## Figures and Tables

**Table 1:** Face types according to facial index.

Face Type	Facial Index
Euryprosopic (broad face)	≤84.9
Mesoprosopic (round face)	85.0–89.9
Leptoprosopic (long face)	≥90.0

**Table 2:** The basic descriptive statistics of this study’s sample: number (N), mean age in years with standard deviation (SD).

Variables Gender	N (%)	Age (Mean ± SD)
male	43 (43)	16.7 (1.3)
female	57 (57)	17.9 (4.2)
Total	100 (100)	17.5 (3.4)

**Table 3:** Face measured values according to gender.

Variables	Gender	N	Mean ± SD	95% CI Lower–Upper	*p* Value
Face length (cm)	male	43	11.6 ± 0.8	−0.0–0.7	0.071
	female	57	11.2 ± 1.1		
Face width (cm)	male	43	11.7 ± 0.8	−0.3–0.9	<0.05
	female	57	11.1 ± 0.6		
Facial index	male	43	99.1 ± 8.4	−6.3–2.1	0.323
	female	57	101.2 ± 11.9		

**Table 4:** Face type distribution according to gender.

Variables Gender	Euryprosopic Face N (%)	Mesoprosopic Face N (%)	Leptoprosopic Face N (%)	Total N (%)
male	2 (2)	3 (3)	38 (28)	43 (43)
female	3 (3)	6 (6)	48 (48)	57 (57)
Total	5 (5)	9 (9)	86 (86)	100 (100)

**Table 5:** Face type and gender *p* values.

Face Types	Gender	N	Value	df	*p* Value
Euryprosopic face	male	2	0.411	2	0.814
	female	3			
Mesoprosopic face	male	3			
	female	6			
Leptoprosopic face	male	38			
	female	48			

**Table 6:** Tooth measurements of both genders.

Variables	Gender	N	Mean ± SD	95% CI Lower–Upper	*p* Value
Tooth length, left (mm)	male	43	10.2 ± 1.1	−0.2–0.7	0.290
	female	57	9.9 ± 1.0		
Tooth width, left (mm)	male	43	9.0 ± 0.7	−0.1–0.4	0.363
	female	57	8.8 ± 0.7		
Tooth length, right (mm)	male	43	10.1 ± 1.2	−0.2–0.7	0.216
	female	57	9.8 ± 0.7		
Tooth width, right (mm)	male	43	8.9 ± 0.6	−0.1–0.4	0.380
	female	57	8.8 ± 0.7		
Tooth index, left	male	43	88.9 ± 11.8	−5.5–3.5	0.661
	female	57	89.9 ± 10.9		
Tooth index, right	male	43	89.7 ± 11.7	−5.1–3.8	0.767
	female	57	90.3 ± 10.8		

**Table 7:** Comparison of tooth and facial indices between genders.

Variables	Gender	N	Mean ± SD	95% CI Lower–Upper	*p* Value
Tooth index vs. facial index	male	43	−9.8 ± 14.0	−12.8–(−6.8)	<0.001
	female	57	−11.3 ± 16.0	−14.2–(−8.3)	<0.001

## Data Availability

The data presented in this study are available upon request from the corresponding author.
